# Investigation of racial differences in survival from non-small cell lung cancer with immunotherapy use: A Texas study

**DOI:** 10.3389/fonc.2022.1092355

**Published:** 2023-01-09

**Authors:** Olajumoke A. Olateju, Zhen Zeng, Oluwasanmi O. Adenaiye, Tyler J. Varisco, Marjan Zakeri, Sansgiry S. Sujit

**Affiliations:** ^1^ Department of Pharmaceutical Health Outcomes and Policy, University of Houston College of Pharmacy, Houston, TX, United States; ^2^ Department of Medicine and Rehabilitation Science, University of Pittsburgh Medical Center, Pittsburgh, PA, United States

**Keywords:** immunotherapy, non-small cell lung cancer, racial disparity, retrospective study, survival analysis

## Abstract

**Background:**

The use of immunotherapy is associated with improved survival among patients with Non-Small Cell Lung Cancer (NSCLC) and has gained widespread use in its management. However, there is limited information on whether the survival benefits associated with immunotherapy differ among races and ethnicities.

**Objective:**

This study aimed to investigate racial differences in survival amongst patients with NSCLC who received immunotherapy as the first-line treatment in Texas.

**Methods:**

Patients with NSCLC who received immunotherapy between October 2015 to December 2018 were identified from the Texas Cancer Registry (TCR). Disease-specific survival was evaluated and compared among patients across racial/ethnic categories using the Kaplan-Meier survival analysis, log-rank test, and a multivariable Cox proportional hazard regression model following an inverse probability treatment weighting (IPTW) propensity score analysis.

**Results:**

A total of 1453 patients were included in the analysis. Median survival (in months) was longest among Asians (34, 95% CI: 15-Not Estimable), followed by African Americans (AAs) (23, 95% CI: 15-34), Hispanics (22, 95% CI: 16-26), and Whites (19, 95% CI: 17-22). The adjusted regression estimates had no statistically significant differences in survival among AAs (aHR = 0.97; 95% CI = 0.78-1.20; P =0.77) and Hispanics (aHR = 0.96; 95% CI = 0.77-1.19, P =0.73) when compared to White patients. Asians on the other hand, had 40% reduction in mortality risk compared to Whites (aHR = 0.60; 95% CI = 0.39-0.94, P = 0.03).

**Conclusions:**

Our study indicated that African Americans and Hispanics do not have poorer survival compared to White patients when receiving immunotherapy as first-line treatment. Asians however had longer survival compared to Whites. Our findings suggest that existing racial disparity in NSCLC survival might be mitigated with the use of immunotherapy and should be considered in providing care to these minority groups.

## 1 Introduction

Non-small cell lung cancer (NSCLC) is the most common type of lung cancer in the United States (US) and accounts for about 85% of all lung cancer cases (1, 2). Lung cancer is the leading cause of cancer-related deaths in the U.S (3). Fortunately, lung cancer-related mortality is on the decline, largely due to advances in treatment options (4, 5). Immunotherapy is an innovative therapy that has been well documented to improve survival in patients with NSCLC; these drugs act by activating immune cells and enhancing their antitumor responses (6). In the past decade, the U.S. Food and Drug Administration (FDA) has approved immunotherapeutic agents including immune checkpoint inhibitors (ICIs) and cytotoxic T-lymphocyte–associated protein 4 receptor (CTL4-A) inhibitors (7) as first- and second-line agents for NSCLC (7, 8).

Many studies have reported that racial disparity exists in lung cancer (8–13). For instance, across racial groups in the U.S., AAs have the highest incidence of lung cancer and mortality rates despite having lower smoking prevalence compared to Whites (14, 15). The American Lung Association (ALA) reports on racial differences among Asians, African Americans, Hispanics, and Whites with regards to prevalence, access to treatment and survival; ALA’s statistics have shown poorer survival among African Americans and Hispanics compared to their White counterparts even when there is access to treatment (16). Further complicating this issue is the underrepresentation of minority racial groups in clinical trials targeting cancers (17). Many trials that have led to the approval of several immunotherapy drugs for NSCLC did not consider national representation of racial groups or racial differences in the burden of the disease in their study samples (18–22). These trials did not consider oversampling the minority groups to support subgroup analyses. In these trials, African American patients comprised only 1-4% of the treatment and control samples, despite the fact that 13.6% of the US population are AAs (23). Just as concerningly, Hispanics were not considered as a distinct category despite their increasing representation, currently at 18.9% of the U.S. population (23). Only few observational studies have evaluated immunotherapy for NSCLC in diverse patient samples found in real-world, clinical settings (7, 24–26) and although these studies provided valuable insights into whether racial disparity occurs with immunotherapy utilization, they were limited by sample size, insufficient representation of minority races or ethnic groups, and low generalizability. To our knowledge, no state-specific study has been done as the majority of previous studies were retrospective reviews of patients receiving treatments in a single treatment center (7, 27). Based on the ALA statistics, the state of Texas is below the national average in achieving racial equity for Lung cancer and White patients often have higher survival rates (28). As such, we hypothesized that White patients with NSCLC have longer survival compared to minority races when immunotherapy is received as the first course of therapy in Texas. Therefore, the objective of this study was to examine if there are racial differences in survival from NSCLC when immunotherapy is administered as the first course of treatment among patients receiving treatment in Texas.

## 2 Methods

### 2.1 Study design and data source

Our study was a retrospective cohort study of patients with histologically confirmed NSCLC who received immunotherapy as their first-line of treatment. Patient data were obtained from the Texas Cancer Registry (TCR) database. The TCR is a statewide population-based cancer registry with gold certification by the North American Association of Central Cancer Registries and is recognized as one of the largest cancer registries in the United States (29). The database provides information on cancer patients’ sociodemographic, tumor, and other clinical characteristics as well as the general class of treatments received as the first-line of treatment (30). All study procedures were approved by the University of Houston Institutional Review Board (IRB) with a waiver of informed consent as this was secondary research that used de-identified data. The study followed the Strengthening the Reporting of Observational Studies in Epidemiology (STROBE) reporting guideline (31).

### 2.2 Identification of study population and variables

**Figure 1:**
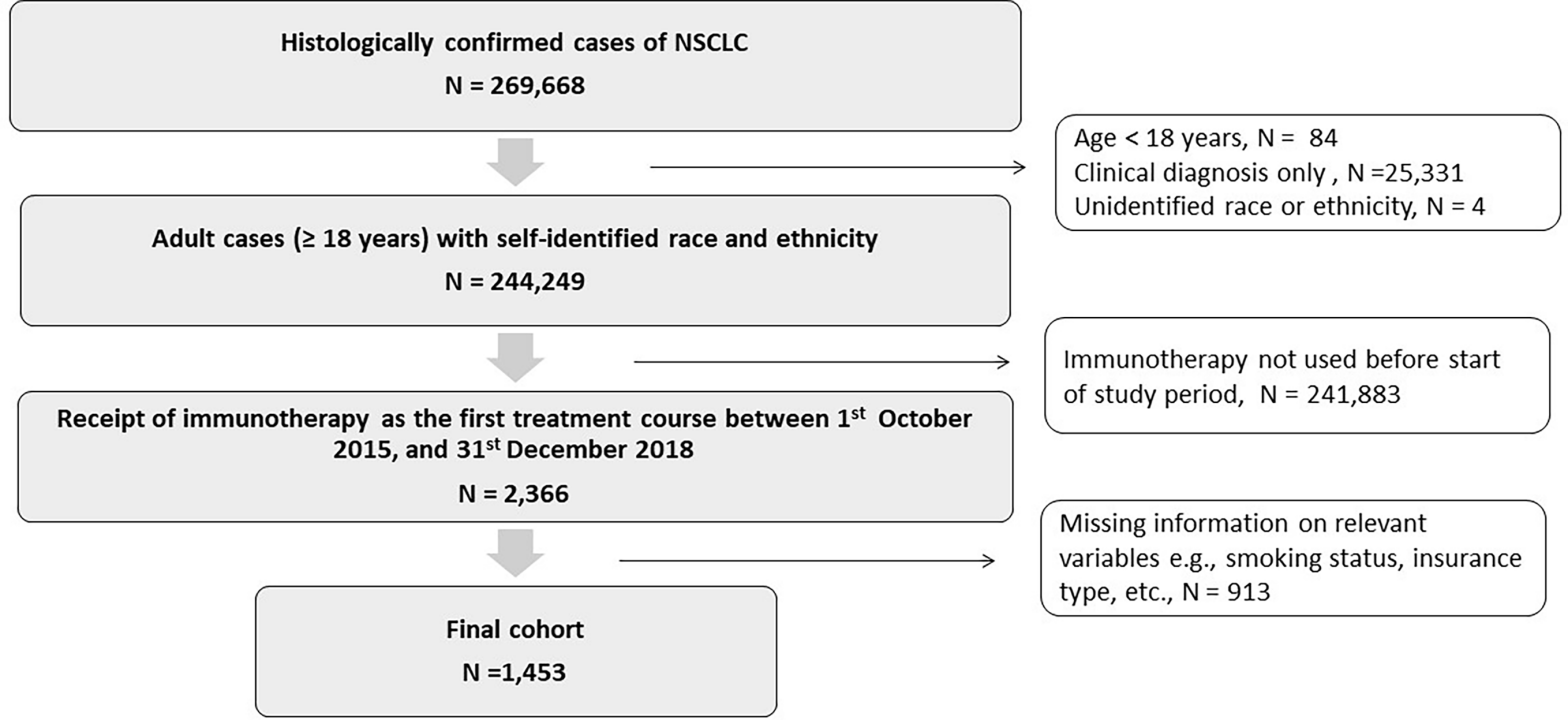
Consort diagram showing cohort selection: Patients 18 years and older with histologically confirmed cases of non-small cell lung cancer who received immunotherapy as the first treatment course between October 1, 2015, and December 31, 2018.

We identified patients with NSCLC, aged 18 years and above, who received immunotherapy as the first-line of treatment from October 2015 to December 2018. Patients were retrospectively followed till December 2020. Patients were excluded if they had any missing values for race, ethnicity, or any other study variables (**Figure 1**). The third edition of the International Classification of Diseases for Oncology (ICD-O) codes was used to identify cases of NSCLC, based on the primary site of the cancer, its morphology, and behavior (**Table S1**) (30, 32).

The primary independent variable was race and ethnicity, referred to simply as race henceforth in the study. The use of immunotherapy was defined as whether patients received an immunotherapy agent as the first-line of treatment. The cancer stage was classified as localized, regional, and distant, according to the classification by the Commission on Cancer (CoC) (31, 33). The primary exposure was the receipt of immunotherapy, defined as being treated with immunotherapy as first-line of treatment. The unexposed group did not receive immunotherapy as first-line agents but received other treatments such as chemotherapy. The primary outcome was disease-specific survival and was defined as the time from initiation of immunotherapy to death or censoring at end of the follow-up period. Censored patients were those who were alive or died of other causes during the study period (34).

Covariates evaluated were demographic and socioeconomic characteristics such as age, sex, insurance type, county-level poverty index, and geographical location. Patient’s smoking history and clinical characteristics such as cancer stage were also identified and defined at baseline. Immunotherapy is commonly administered with other agents, especially chemotherapy (29, 32), so receipt of other treatment modalities as first-line agents such as chemotherapy, radiotherapy, hormone therapy, and surgery were also measured.

### 2.3 Statistical analysis

Data management and statistical analyses were performed using SAS v9.4 software (SAS Institute Inc., Cary, NC, USA). Two-sided statistical significance was used to test hypotheses and was defined as P<0.05.

### 2.4 Descriptive statistics

To describe the baseline characteristics of the study cohort, continuous variables were presented as means with standard deviations, while categorical variables were presented as frequency and percentages, and comparisons were made using the Chi-square test. Comparisons across racial categories were performed using the analysis of variance (ANOVA) test.

### 2.5 Survival analysis

Crude survival differences across racial categories were obtained using unadjusted Kaplan-Meier (K-M) analysis and a bivariate Cox proportional hazards (PH) regression model. K-M curves were used to depict the monthly survival probabilities of patients per racial category and statistical differences in these survival probabilities across the study period were determined using the log-rank test. Pairwise analysis of the K-M analysis was done to evaluate survival differences in each minority group versus Whites alone while adjusting for multiplicity hence inflation of Type 1 error using the Sidak test (35). Sidak adjustment assumes that each comparison is independent of the others and has more power than the more conservative Bonferroni adjustment (36). Median survival times obtained from the K-M analysis were compared across races. As a secondary analysis to obtain survival time, the restricted mean survival time (RMST) was obtained for all races. The RMST is not commonly used in survival analysis but is generally considered a more reliable estimate than the median survival time estimated using the K-M curve due to its ability to circumvent skewness and challenges associated with censoring in survival data (37, 38). In addition, it provides a summary of the survival time in the entire period of observation, as opposed to obtaining the survival rate at a specified time as with the K-M curve (38).

A multivariable Cox proportional hazards regression model was fitted to compare mortality risk among the different racial categories while adjusting for baseline covariates. Cancer stage and smoking status of the patients were introduced as interaction terms in the Cox proportional hazard model to examine if the survival of patients across racial categories varied according to the spread of cancer or smoking history (2, 39).

### 2.6 Propensity score analysis

The difference in mortality risk between patients across racial and ethnic groups was further examined after balancing differences in treatment (racial) groups by inverse probability treatment weighting (IPTW). IPTW has the advantage of yielding marginal treatment estimates while conserving sample size of all propensity score (PS) methods (40). Multiple PS technique which controls for bias by comparing more than two treatment groups was used in this study as there were four racial groups (41). The IPTW is the probability of assignment to each treatment category (41). Pairwise PS analysis when dealing with more than two groups i.e., comparing two groups at a time, is not recommended because the probability of choosing all treatment groups will be greater than one, and the model fits are less efficient leading to variance inflation (41, 42) hence our opting for the multiple propensity score technique. To carry out our PS analysis, PS (probability of each patient of a particular race belonging to another racial group) was obtained using a multinomial logistic regression analysis (41, 43). The generalized logit function was specified in the link option to contrast minority races to White as the reference group. All identified potential confounders, including interaction terms, were added to the logistics model. Overlap of generated PS across the racial groups was assessed. The inverse of the propensity scores was then used to generate the IPTW, also known as the propensity score weights (28, 29). The weights were stabilized to prevent undue influence of extreme weights which can bias the result (40). The balance of the baseline characteristics across treatment groups was assessed using Absolute Standardized Mean Differences (ASMD) (40, 44). Finally, a weighted multivariable Cox proportional hazard model was fitted with race as the only predictor and White patients as the reference group.

### 2.7 Sensitivity analysis

Sensitivity analysis was performed to examine the possible impact of informative censoring on the results obtained (44, 45). First, the risk of mortality was evaluated with censored patients assumed to have been observed for the entire follow-up period, i.e., the entire period of observation. This analysis tests the hypothesis that censored cases are at low mortality risk and thus have more extended times of death from NSCLC than other cases (45). A second analysis was done whereby patients who died of causes other than NSCLC were not censored. This analysis tests the hypothesis that people who died of other causes (and thus censored) would have experienced this if they had not died and were thus at high risk of mortality from NSCLC (45). The sensitivity analysis was repeated using the IPTWs and race as the only covariate.

## 3 Results

### 3.1 Characteristics of the study cohort

A total of 244,249 adult patients with NSCLC diagnosis and self-reported race and ethnicities were identified in the TCR database. The racial categories were defined as non-Hispanic White, non-Hispanic African American or African American, non-Hispanic Asian and Hispanic. These are henceforth referred to as White, AA, Asian, and Hispanic respectively. Of the identified patients with NSCLC, 2366 received immunotherapy as the first-line of therapy between October 2015 and December 2018. After excluding patients with missing information, the final cohort consisted of 1,453 patients. An attrition flowchart detailing inclusion and exclusion criteria is provided in **Figure 1**.

Among the study population, 1,044 (71.8%) were White, 185 (12.7%) were African American, 172 (11.8%) were Hispanic, and 72 (3.6%) were Asian. The median age for all patients was 68 years (Interquartile range, IQR: 61 – 74 years) [White: 69 (IQR: 27 - 98) years, African American: 66 (IQR: 40 - 90) years, Hispanic: 65 (IQR: 21 - 89) years, and Asian: 63 (IQR: 36 - 89) years]. All patients had immunotherapy initiation within two months of NSCLC diagnosis. More than half of the study population were females (52.6%). A larger proportion of all patients had government-type insurance (66.8%), lived in metropolitan or urban areas (85.1%), were current or former smokers (80.3%), had metastatic cancer (75.6%), and received chemotherapy (as adjunct therapy or second-line treatment; 64.6%). Asians were the youngest population, with 44.2% of them being younger than 65 years of age at the diagnosis. Hispanics had the highest proportion (11.1%) of uninsured patients. African Americans (43.2%) and Hispanics (40.7%) had the highest proportion of patients at the highest percentile of the census tract poverty level category (20 – 100%), considered as the most extreme level of poverty. Almost all the Asian population lived in metropolitan areas (98.1%). The rate of smoking was highest among African Americans (86.5%) and Whites (83.0%) followed by Hispanics (64.0%) and Asians (59.6%).

### 3.2 Unadjusted survival characteristics of patients across racial groups

**Figure 2:**
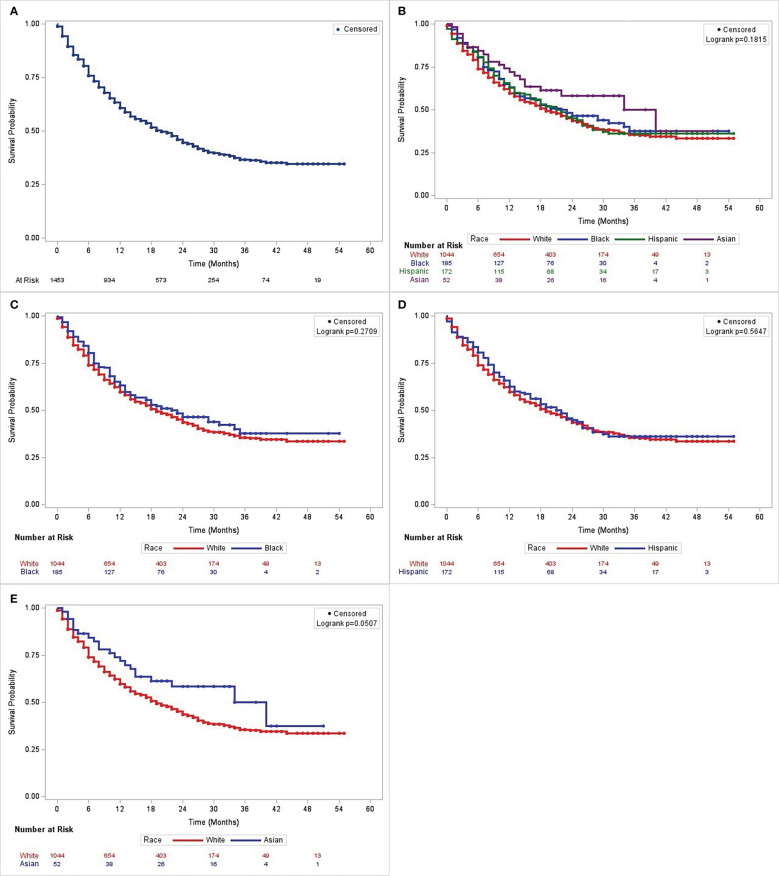
Kaplan-Meier curves showing disease-specific survival in patients with non-small cell lung cancer who received immunotherapy as the first course of treatment in Texas from 2009 to 2018. From L to R: **(A)** the entire cohort; **(B)** patients stratified by race and ethnicity; and comparisons between **(C)** African American and White patients, **(D)** Hispanic and White patients, and **(E)** Asian and White patients.

A larger proportion of White patients (N = 588; 56.3%) died during the observation period (**Table S1**). This was followed by Hispanics [N = 95; 55.2%], African Americans [N= 89; 48.2%] and Asians [N= 52; 42.3%]. While Asians maintained a slightly higher survival across the follow-up period, the overall Kaplan-Meier estimates (**Figure 2**) did not show any significant differences across all racial groups (P = 0.18). The pairwise tests showed that Asians had a higher survival probability compared to Whites (P = 0.05) (**Figure 2E**). The cohort’s overall median disease-specific survival (DSS) was 19 months (95% CI = 17 - 22 months) and Asians had the longest median survival time (34 months, lower bound 95% CI = 15 months), followed by African Americans (23 months, 95% CI = 15 - 34), Hispanics (22 months, 95% CI = 16 - 26), and Whites (19 months, 95% CI = 17 - 22). The upper confidence interval limit of the median DSS for Asians was inestimable due to high rate of censoring in this population (30). The RMST values [(mean (SD)] obtained were 25.4 (0.68) months for Whites, 27.3 (1.64) months for African Americans, 26.3 (1.64) months for Hispanics, and 30.8 (3.14) months for Asians. (**Figure S1**). As with the K-M analysis, there was no statistically significant difference in survival among the racial categories (P = 0.29). No pairwise analysis was performed for the RMST analysis.

### 3.3 Adjusted association between race and survival using conventional Cox proportional hazard regression

**Figure 3:**
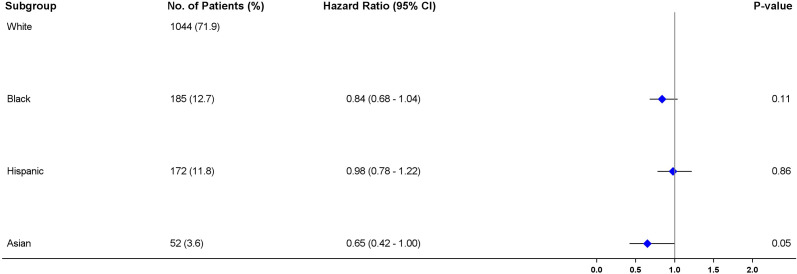
Forest plot of multivariable Cox proportional hazard regression analysis showing the association between patient characteristics and disease-specific survival using regression adjustment.

The multivariable Cox proportional hazard regression results (**Figure 3**) indicated no significant differences in survival of African American (aHR = 0.84; 95% CI = 0.68-1.04, P =0.113) patients, and Hispanic patients (aHR = 0.98; 95% CI = 0.78-1.22, P =0.862) in comparison with White patients when immunotherapy was administered as the first-line of treatment. Asians on the other hand, had about 35% reduction in risk compared to White patients but the upper confidence level shows evidence of probability of similar survival chances as White patients (aHR = 0.65, 95% CI = 0.42-1.00, P =0.05) (52,53). Although cancer stage and smoking status were independently associated with survival in the adjusted regression model (P<0.05), their interaction terms were not statistically significant (P>0.05), indicating that the influence of race on survival does not differ by the stage of cancer or smoking status when immunotherapy is received.

The results of the first sensitivity analysis which assumed that censored observations had the longest follow-up period provided similar results with the main analysis (African Americans: aHR = 0.86, 95% CI = 0.69 -1.07, P =0.18, Hispanics: aHR = 1.01, 95% CI = 0.81 - 1.26, P =0.97; Asians: aHR = 0.64, 95% CI = 0.42 – 0.99, P =0.04). The second analysis which assumed that patients who died from other causes had similar mortality risk as those who died from cancer, did not show any significant difference among the racial groups regarding the mortality risk.

### 3.4 Propensity score estimation

**Figure 4:**
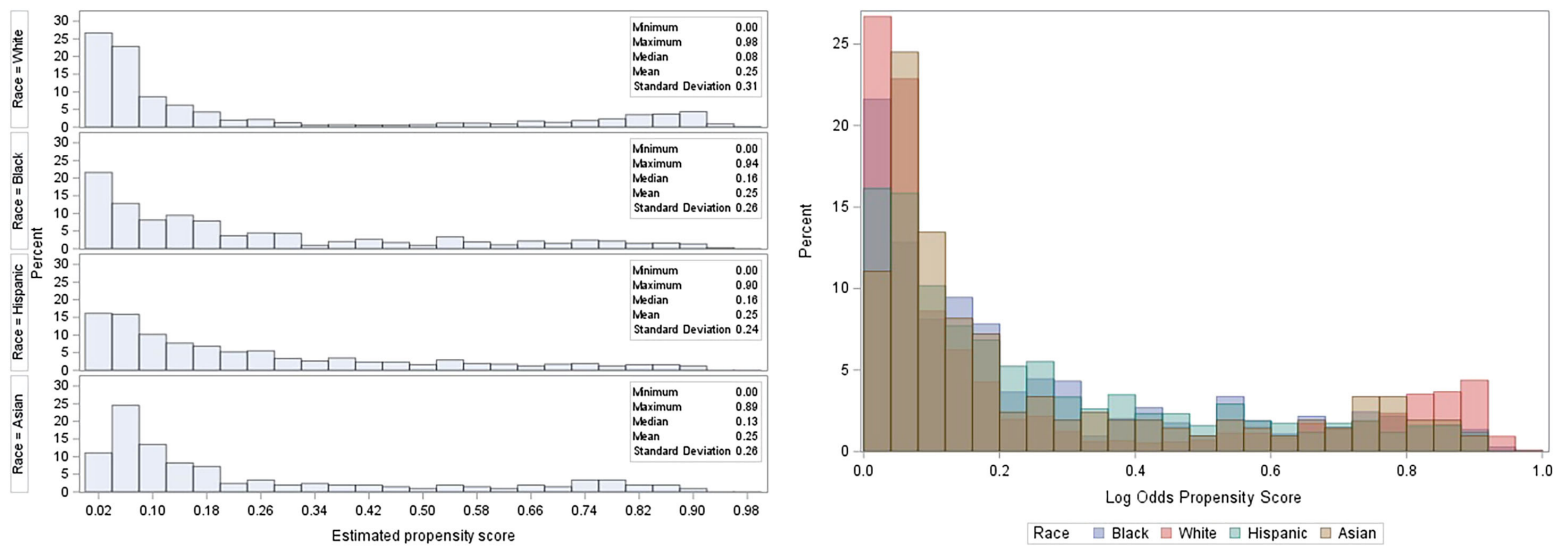
Histogram showing the distribution of propensity scores by treatment (racial) group. From L to R: stacked histogram showing individual propensity scores for treatment groups, the overlap of propensity scores among treatment groups.

**Figure 5:**
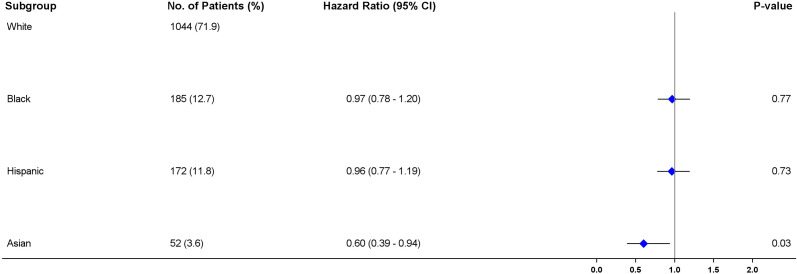
Forest plot of multivariable Cox proportional hazard regression analysis showing the association between patient characteristics and disease-specific survival using the inverse probability treatment weighting (IPTW) method.

The baseline characteristics among all races were comparable after adjustment using IPTW. There was considerable overlap among the treatment groups (**Figure 4**). The distribution of propensity scores was similar between all racial groups and the groups were thus comparable (31). The maximum ASMD for all covariates after IPTW was 11% and 90% of the measured covariates after IPTW had ASMD values below 0.1, as shown in (**Table 1**; **Figure S2**). This value was much less than the 25% recommended value (33, 34) confirming a good balance across the racial groups. ASMD below 0.1 indicated an acceptable balance between treatment (racial) groups (35). **Figure 5** shows the regression estimates for the PS analysis. The propensity-score-weighted Cox proportional hazards model showed similar results with Cox analysis using regression adjustment (**Figure 5**). African Americans and Hispanics had comparable mortality risk as White patients (African Americans: aHR = 0.97, 95% CI = 0.78-1.20, P = 0.77, Hispanics: aHR = 0.96, 95% CI = 0.77-1.19, P = 0.73) while Asians had lower mortality risk compared to White patients (aHR = 0.60, 95% CI = 0.39 - 0.94, P = 0.03). The results of the sensitivity analyses using IPTW was similar to the results obtained from the adjusted regression model.

**Table 1:** Descriptive characteristics of 1453 Patients with Non-Small Cell Lung Cancer Identified in the Texas Cancer Registry from October 2015 to December 2018 and Absolute Standardized Mean Differences (ASMD) Before and After Propensity Score Analysis using the Inverse Probability Treatment Weighting (IPTW) Method.

		Race					ASMD	
	Total	White	African American	Hispanic	Asian			
Characteristic	(N =1453)	(N =1044)	(N=185)	(N=172)	(N=52)	P-value	Before IPTW	After IPTW
**Time to treatment initiation, Median (Q1-Q3), month**	1.0 (1.0 – 2.0)	1.0 (1.0 – 2.0)	1.0 (1.0 – 2.0)	1.0 (1.0 – 2.0)	1.0 (1.0 – 2.0)	0.6	0.09	0.04
Age group								
18-64	532 (36.6)	344 (32.9)	80 (43.2)	79 (45.9)	29 (55.8)	<0.0001*	0.27	0.06
≥65	921 (63.4)	700 (67.1)	105 (56.8)	93 (54.1)	23 (44.2)			0.07
Sex								
Male	689 (47.4)	506 (48.5)	87 (47.0)	74 (43.0)	22 (42.3)	0.50	0.10	0.08
Female	764 (52.6)	538 (51.5)	98 (53.0)	98 (57.0)	30 (57.7)			0.09
Insurance								
Private	401 (27.6)	280 (26.8)	44 (23.8)	53 (30.8)	24 (46.2)	<0.0001*	0.10	0.06
Government	971 (66.8)	716 (68.6)	132 (71.3)	100 (58.1)	23 (44.2)		0.21	0.07
Uninsured	81 (5.6)	48 (4.6)	9 (4.9)	19 (11.1)	5 (9.6)		0.23	0.08
Poverty Index								
0-<5	272 (18.7)	231 (22.1)	15 (8.1)	12 (7.0)	14 (26.9)	<0.0001*	0.40	0.06
5-9.9	352 (24.2)	292 (28.0)	22 (11.9)	27 (15.7)	11 (21.2)		0.37	0.07
10-19.9	507 (34.9)	357 (34.2)	68 (36.8)	63 (36.6)	19 (36.5)		0.05	0.06
20-100	322 (22.2)	164 (15.7)	80 (43.2)	70 (40.7)	8 (15.4)		0.54	0.03
Location								
Metro	1236 (85.1)	866 (83.0)	162 (87.6)	157 (91.3)	51 (98.1)	0.0008*	0.23	0.11
Non-metro	217 (14.9)	178 (17.1)	23 (12.4)	15 (8.7)	1 (1.9)			0.10
Smoking status								
Never smoked	286 (19.7)	178 (17.0)	25 (13.5)	62 (36.0)	21 (40.4)	<0.0001*	0.43	0.08
Current/former smoker	1167 (80.3)	866 (83.0)	160 (86.5)	110 (64.0)	31 (59.6)			0.06
Stage								
Localized	82 (5.6)	58 (5.6)	9 (4.9)	14 (8.1)	1 (1.9)	0.17	0.06	0.06
Regional	273 (18.8)	205 (19.6)	33 (17.8)	31 (18.1)	4 (7.7)		0.08	0.09
Distant	1098 (75.6)	781 (74.8)	143 (77.3)	127 (73.8)	47 (90.4)		0.11	0.07
Histopathology								
Non-squamous cell	1184 (81.5)	833 (79.8)	154 (83.2)	150 (87.2)	47 (90.4)	0.03*	0.23	0.05
Squamous cell	269 (18.5)	211 (20.2)	31 (16.8)	22 (12.8)	5 (9.6)			0.05
Surgery								
No	1373 (94.5)	982 (94.1)	174 (94.0)	165 (95.9)	52 (100.0)	0.24	0.08	0.07
Yes	80 (5.5)	62 (5.9)	11 (6.0)	7 (4.1)	0 (0.0)			0.08
Radiotherapy								
No	908 (62.5)	640 (61.3)	119 (64.3)	118 (68.6)	31 (59.6)	0.28	0.15	0.11
Yes	545 (37.5)	404 (38.7)	66 (35.7)	54 (31.4)	21 (40.4)			0.10
Hormone therapy^†^								
No	1426 (98.1)	1026 (98.3)	183 (98.9)	167 (97.1)	50 (96.1)		0.08	0.05
Yes	27 (1.9)	18 (1.7)	2 (1.1)	5 (2.9)	2 (3.9)			0.07
Chemotherapy								
No	514 (35.4)	383 (36.7)	57 (30.8)	55 (32.0)	19 (36.5)	0.34	0.11	0.06
Yes	939 (64.6)	661 (63.3)	128 (69.2)	117 (68.0)	33 (63.5)			0.05

Number and percentages are reported except stated otherwise.

^†^Hormone therapy did not meet the Chi-square assumption that the expected value of cells in the contingency table should be 5 or greater in at least 80% of cells.

*Statistically significant at a significance level of 5%.

ASD means absolute standardized difference, the largest ASD among treated groups is reported.

## 4 Discussion

This retrospective cohort study examined if differences in survival exist amongst NSCLC patients of different races who receive immunotherapy as the first-line of treatment in Texas. Our findings revealed no differences in survival among Whites, African Americans, and Hispanics (P>0.05), while Asians had a 40% decreased risk of mortality. We adjusted for patient baseline characteristics using regression adjustment and propensity score analysis, and there was no qualitative difference in the results obtained *via* both methods, as both methods gave the same interpretation of mortality risk across the racial groups. We demonstrated that the propensity score analysis reduced bias from measured confounders based on ASMD values, improving the reliability of our estimates.

Our findings are similar to those reported in previous studies.A retrospective cohort study using data collected from over 260 community cancer clinics in the U.S (25). reported a longer median overall survival (OS) among Asians [9.7 (IQR: 6.8 –13.2)] followed by African Americans [9.0 months (IQR: 4.8-12.7)], and Whites [8.0 months (IQR: 7.3 -9.2)]. This pattern was observed in our study, but they could not make further comparisons because their study did not include the Hispanic population, and race-stratified multivariable analysis was not done. Another study evaluated differences in survival between White and African American patients receiving treatment at a single institution in Georgia state (27) and reported similar OS (aHR = 0.90, 95% CI = 0.59 - 1.37, P = 0.13) and progression-free survival, PFS (aHR = 2.70, 95% CI = 1.08 – 6.74, P = 0.08) among the groups. This is also similar to our finding in which there was no significant difference in DSS between African American and White patients, but again other minority races were not considered in their study. Ayers et al. (7) oversampled African American patients (30.1% of the racial cohort) and included Asian (9.6%) and Hispanic populations (14.1%) in their study. Similar to our finding, their multivariable Cox analysis found no significant difference in survival of African Americans (aHR = 0.60, 95% CI = 0.34-1.03, P = 0.06) and Hispanics (aHR = 0.69, 95% CI = 0.37-1.27, P = 0.23) when compared to Whites, while their Asian population had improved overall survival (aHR = 0.32, 95% CI = 0.12-0.85, P = 0.02). After PS analysis with White and African American patients, African Americans showed improved survival, but the strength of the association was weak (aHR = 0.53, 95% CI = 0.28-1.01, P = 0.054). Our study findings are in line with previous studies, therefore, suggest that minority races can equally benefit from immunotherapy if they have access to immunotherapy. This is a positive finding since many studies have reported longer survival among only White population using conventional therapies like chemotherapy and surgery (9, 36).

In our analyses, the cancer stage, and patients ‘smoking history were considered as potential moderators of the effect of race on survival. This is because these variables are known independent predictors of survival from lung cancers, individual responses to immunotherapy may therefore differ based on these factors’ asides from racial status (37–39). In addition, race mediated through genetics may influence the aggressiveness of a cancer, hence, the stage (40) and behavioral factors such as smoking habits may differ across races. For instance, in our study population, there were more White and African American smokers than Hispanics and Asians who smoked. Our analysis however indicated no interaction between race and cancer stage, or between race and smoking status. Therefore, we did not proceed with subgroup analysis for these variables to prevent inflation of type 1 error rates (31). Our analysis also included an evaluation of how sensitive our Cox regression estimates were to possible informative censoring, and the results showed that our study findings were moderately robust to informative censoring. The fact that Asians lost their “superior” survival in the second sensitivity analysis may suggest that Asians who died of other causes were systematically different from those who died of lung cancer. Also, high censoring percentage which was observed with Asians (57.7%) can increase bias if patients who provided most of the information, were censored (41). Overall, this result still shows that patients of all races may equally benefit from immunotherapy for treatment of NSCLC (37, 62–64)

The strength of our study is in its ability to corroborate existing but limited knowledge about racial variations in survival that may exist with immunotherapy utilization. To further improve on these studies, we used more rigorous analytic techniques and included more populations utilizing this therapy in real-world. For the first time in related studies, we used multiple propensity score techniques to effectively reduce potential confounding bias through its pseudo-randomization (31, 44) and increased the reliability of our estimates. PS methods have been mainly used for two treatment groups. Another strength of our study was balancing pre-treatment characteristics for more than two groups, which is not very common. Other studies did not conduct sensitivity analysis and to our knowledge, our study has the largest sample size (N =1,453) and explored the most heterogeneous patient population due to utilization of a registry database collecting information from all cancer institutes in Texas (8–12, 36). The distribution of racial categories in our study was more representative of national estimates than previous studies and clinical trials. The implication of our overall study finding is that the efficacy of immunotherapy as observed in clinical trials is likely realized in the heterogenous patient population in real world, especially across an important social and or biologic construct such as race. Access to immunotherapy should be increased for minority races since disparity in access to treatment has been reported by NSCLC (42, 43).

Our study has limitations. Firstly, we did not have information on some parameters such as the specific immunotherapeutic agents used by the patients, their dosing regimen, and duration of therapy. Response markers to immunotherapy and tumor mutations that could have been used for prediction of a patient’s response to immunotherapy, were also not available (6). Second, Race and ethnicity were self-reported; information on ancestry and genetic data may provide more accurate information on the survival characteristics (46). The database we used lacked genetic data which may also drive differential survival characteristics observed in our study, for instance, being Asian has been reported to be a favorable prognostic factor for overall survival in NSCLC irrespective of smoking status (44). Our Asian population was small, similar to previous studies. This might be due to the low incidence of lung cancer among Asian population (16, 45). Third, unmeasured baseline characteristics which may act as confounders e.g., comorbidities were largely missing and were not considered in our analysis. Also, we excluded patients with missing information, which might have resulted in biased findings. Lastly, given that this study only focused on Texans, a more robust study in a nationally representative population is needed to confirm existing findings.

## 5 Conclusion

This retrospective cohort study showed no differences in survival between African American, Hispanic, and White patients in Texas when immunotherapy was used as the first-line of treatment for NSCLC. These results corroborate findings in previous studies and demonstrated similar outcomes for immunotherapy across races, thus reinforcing the value of observational studies in contributing to evidence-based knowledge and clinical decisions. It is recommended that access to immunotherapy is maintained across minority groups and nationally representative studies being conducted to generalize the finding across U.S. populations.

## Data availability statement

The data analyzed in this study is subject to the following licenses/restrictions: The TCR dataset is available upon request at no cost. Requests to access these datasets should be directed to https://www.dshs.texas.gov/tcr/data/requests.aspx.

## Ethics statement

All study procedures were approved by the University of Houston Institutional Review Board (IRB) with a waiver of informed consent as this was secondary research that used de-identified data. The study followed the Strengthening the Reporting of Observational Studies in Epidemiology (STROBE) reporting guideline.

## Author contributions

OO, ZZ, and SS conceptualized and designed the study. OO did the methodology, data analysis, data visualization and wrote initial draft of the manuscript. OA supported the study design and analysis. OO, OA, and SS interpreted the results. TV and MZ were involved in manuscript drafts. SS was involved in study supervision, review, and editing of the draft. All authors conducted a critical revision of the manuscript for important intellectual content and approved the submitted version.
