# Supplementary material for: Investigation of racial differences in survival from non-small cell lung cancer with immunotherapy use: A Texas study
Source: Front Oncol. 2023 Jan 9;12:1092355. doi: 10.3389/fonc.2022.1092355 (PMC9869031; doi:10.3389/fonc.2022.1092355)

**Supplementary Content**

**Investigation of Racial Differences in Survival from Non-Small Cell Lung Cancer with Immunotherapy Use in Texas**

Olajumoke A. Olateju, Zhen Zeng, Oluwasanmi Adenaiye, Varisco J. Tyler^1^, Marjan Zakeri^1,^ Sansgiry S. Sujit

^1^Department of Pharmaceutical Health Outcomes and Policy, University of Houston College of Pharmacy, Houston, TX, United States

^2^Department of Medicine and Rehabilitation Science, University of Pittsburgh Medical Center, Pittsburgh, Pennsylvania.

Table S1. Identification of cases of non-small cell lung cancer from the International Classification of Diseases (ICD-O), third edition, first revision coding

Table S2. Survival Characteristics of the Study Population

Table S3. Multivariable Logistic Regression Showing the Association Between Patient Characteristics and Survival

Figure S1. Root Mean Survival Time Curve of the Racial Categories

Figure S2. Plots of Maximum Standardized Mean Difference Obtained for Balance of Measured Covariates Among Comparison groups

**Table S1. Identification of cases of non-small cell lung cancer from the International Classification of Diseases (ICD-O), third edition, first revision coding**

|  | **Variable** | **Codes** |
| --- | --- | --- |
| Primary site | Psite | C339, C340 - C343, C348, C349 |
| Morphology | HistTypeICDO3 | 8012, 8046, 8070, 8140, 8240, 8250, 8560, 9050 |
| Behavior | BehaviorICDO3 | 3 |

**Table S2. Survival Characteristics of the Study Population**

|  | White | African American | Hispanic | Asian |
| --- | --- | --- | --- | --- |
| Number of patients | 1044 | 185 | 172 | 52 |
| Number (%) of patients with event | 588 (56.3) | 96 (51.8) | 95 (55.2) | 22 (42.3) |
| Number (%) of patients censored | 456 (43.7) | 89 (48.2) | 77 (44.8) | 30 (57.7) |
| Median survival time, 95% CI (month) | 19 (17 -22) | 23 (15 – 34) | 22 (16 -26) | 34 (15 – NE) |
| Restricted Mean Survival Time (SD) | 25.4 (0.68) | 27.3 (1.64) | 26.3 (1.64) | 30.8 (3.14) |

*NE, Not Estimable

**Table S3. Multivariable Cox Proportional Hazards Regression Model Showing Association between Patient Characteristics and Mortality**

| **Characteristic** | **Hazard Ratio (95% CI)** | **P-value** |
| --- | --- | --- |
| **Age group** |  |  |
| 18-64 | - |  |
| ≥65 | 1.083(0.908,1.291) | 0.38 |
| **Sex** |  |  |
| Female | - |  |
| Male | 1.24 (1.08- 1.43) | **0.003** |
| **Race** |  |  |
| White | - |  |
| Black | 0.84 (0.68 - 1.04) | 0.11 |
| Hispanic | 0.98 (0.79 - 1.22) | 0.86 |
| Asian | 0.65 (0.42 - 1.00) | **0.05** |
| **Insurance** |  |  |
| Uninsured | - |  |
| Government | 1.44 (1.06 - 1.97) | **0.02** |
| Private | 1.42 (1.20 - 1.67) | **<.0001** |
| **Stage** |  |  |
| Distant | - |  |
| Regional | 0.52 (0.42 - 0.65) | **<0.001** |
| Localized | 0.37 (0.25 - 0.56) | **<.0001** |
| **Surgery** |  |  |
| No | - |  |
| Yes | 0.58 (0.39 - 0.88) | **0.009** |
| **Smoking status** |  |  |
| Nonsmoker | - |  |
| Current or former smoker | 1.33 (1.10 - 1.61) | **0.003** |

P-values in bold display significant results.

- Reference group

Backward selection model was used. Variables controlled for were Age at diagnosis, sex, race, smoking status, insurance type, poverty index, stage, histology, and use of chemotherapy, radiation therapy and surgery

**Figure S1. Root Mean Survival Time (RMST) Curve of the Racial Categories**


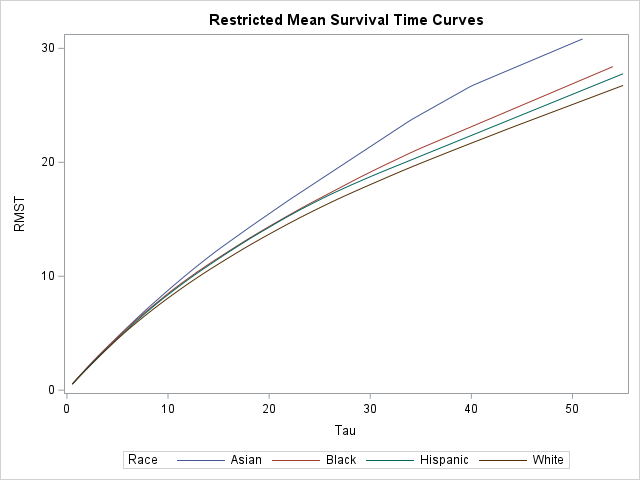


**Figure S2. Plots of Maximum Standardized Mean Difference Obtained for Balance of Measured Covariates Among Comparison groups (White, African Americans, Hispanics, and Asians)**


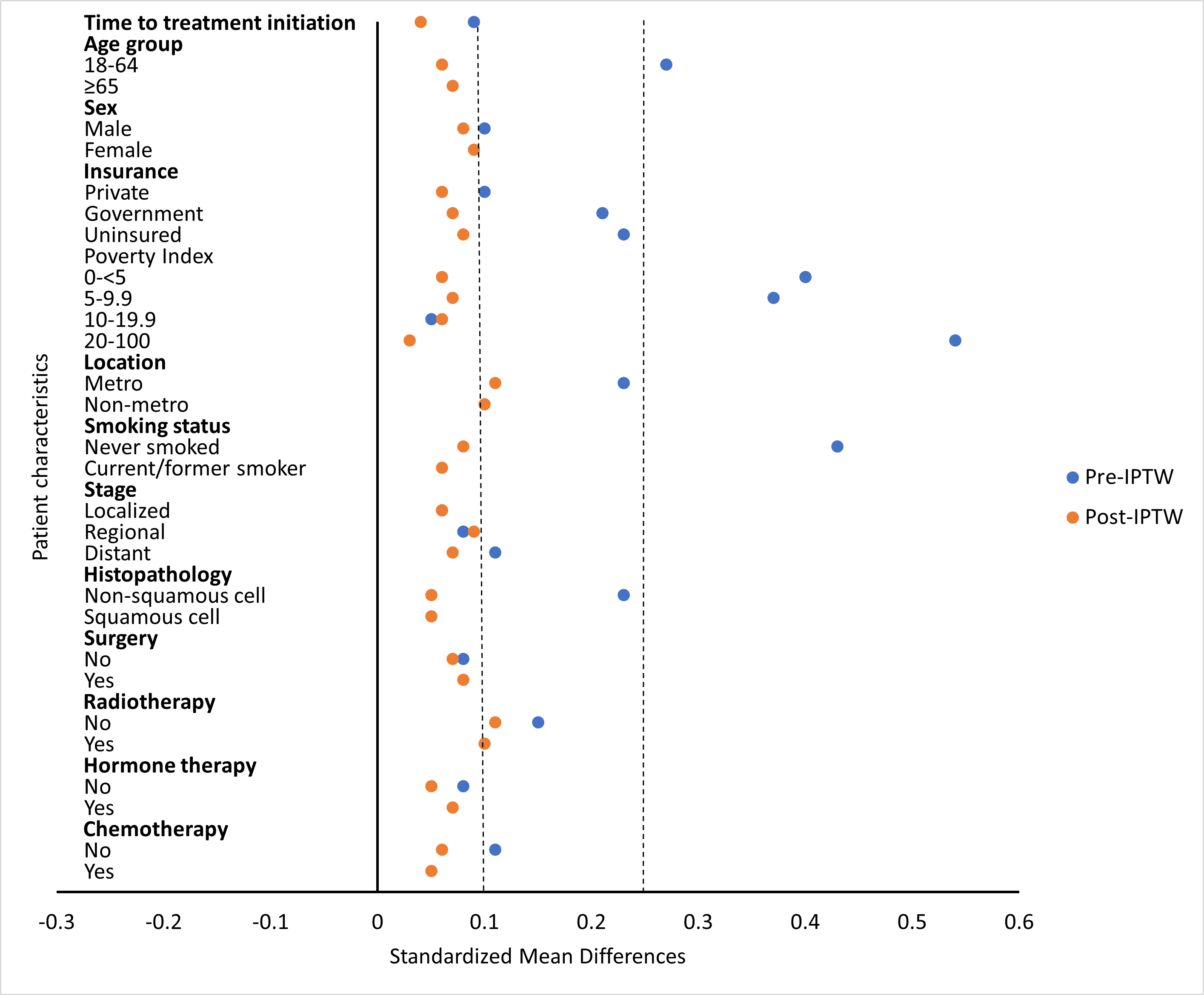

Supplement: Supplementary file 1 [file DataSheet_1.docx]
